# Identification of microspore-active promoters that allow targeted manipulation of gene expression at early stages of microgametogenesis in *Arabidopsis*

**DOI:** 10.1186/1471-2229-6-31

**Published:** 2006-12-21

**Authors:** David Honys, Sung-Aeong Oh, David Reňák, Maarten Donders, Blanka Šolcová, James Andrew Johnson, Rita Boudová, David Twell

**Affiliations:** 1 Laboratory of Pollen Biology, Institute of Experimental Botany ASCR, Rozvojová 135, 165 02 Prague 6, Czech Republic; 2 Department of Plant Physiology, Faculty of Sciences, Charles University, Viničná 5, 128 44, Prague 2, Czech Republic; 3 Department of Biology, University of Leicester, Leicester LE1 7RH, U.K; 4 University of South Bohemia, Faculty of Biological Sciences, Dept. of Plant Physiology and Anatomy, Branišovská 31, 370 05 České Budějovice, Czech Republic; 5 Laboratory of Hormonal Regulations in Plants, Institute of Experimental Botany ASCR, Rozvojová 135, 165 02 Prague 6, Czech Republic

## Abstract

**Background:**

The effective functional analysis of male gametophyte development requires new tools enabling the spatially and temporally controlled expression of both marker genes and modified genes of interest. In particular, promoters driving expression at earlier developmental stages including microspores are required.

**Results:**

Transcriptomic datasets covering four progressive stages of male gametophyte development in *Arabidopsis *were used to select candidate genes showing early expression profiles that were male gametophyte-specific. Promoter-GUS reporter analysis of candidate genes identified three promoters (MSP1, MSP2, and MSP3) that are active in microspores and are otherwise specific to the male gametophyte and tapetum. The *MSP1 *and *MSP2 *promoters were used to successfully complement and restore the male transmission of the gametophytic *two-in-one *(*tio*) mutant that is cytokinesis-defective at first microspore division.

**Conclusion:**

We demonstrate the effective application of MSP promoters as tools that can be used to elucidate gametophytic gene functions in microspores in a male-specific manner.

## Background

The male gametophyte of flowering plants displays a highly reduced structure of two or three cells at maturity and its development provides an excellent system to study many fundamentally important biological processes such as cell polarity, cell division and cell fate determination (reviewed by [1]). An increasing collection of mutations and genes have been characterized that act gametophytically and have been shown to be important for post-meiotic cell division during pollen development in *Arabidopsis*. These include *MOR1/GEM1 *[2] and *TIO *[3], whose functions are essential for regular cell polarity and cytokinesis at first microspore division, termed pollen mitosis I. However, mutations in such essential genes cause gametophytic embryo sac defects and sporophytic lethality [2,3]. This prevents the analysis of homozygous mutations and hinders the functional analysis of their role(s) in specific cell types such as microspores. The native promoters of essential genes such as *TIO *are not useful tools to examine effects of mis-expression of gene or protein domains during pollen development since their broad activities in other sporophytic tissues can be detrimental to early vegetative development prior to gametophytic development. Moreover the use of well characterized male-gametophyte-specific promoters such as LAT52 enables targeted manipulation of gene expression that is restricted to the vegetative cell during pollen maturation after pollen mitosis I ([4]. Therefore we have faced a practical challenge to identify promoters that allow the targeted manipulation of gene expression in microspores.

A number of male-gametophyte-specific promoters that are active at different developmental stages are known. Most data are available for late pollen promoters. These include petunia *chiA *[5], tomato *LAT52 *and *LAT59 *[4,6], rapeseed *Bp10 *[7], maize *Zm13 *[8,9] and tobacco *NTP303 *[10]. In *Arabidopsis *these include the *TUA1 *[11], *AtPTEN1 *[12], *AtSTP6 *[13], *AtSTP9 *[14] and the late vegetative cell-specific *AtVEX1 *[15] promoters. Among these, the tomato *LAT52 *promoter with demonstrated vegetative-cell-specific expression [4,6] was shown to be highly active in number of plant species and has become widely used as a tool to drive pollen-specific expression [16-20]. More recently, promoters active in generative or sperm cells have been identified from lily [21] and *Arabidopsis *[15,22,23].

On the other hand, very few promoters have been identified that are active or specifically active at microspore stage. The tobacco *NTM19 *promoter is the only well characterized promoter that exhibits strict microspore-specific expression with no activity in mature pollen [24,25]. From rapeseed, *Bp4 *mRNA was described as microspore-specific [26], but the Bp4 promoter was later shown to be active only after PMI [24]. However the *BnM3.4 *promoter was active in tetrads and in free microspores [27]. The potato *invGF *promoter is initiated in late microspores and is restricted to the male gametophyte [28]. In *Arabidopsis *available microspore expressed promoters are also limited. The *Arabidopsis *BCP1 promoter is active in microspores and the tapetum [29] while the *AtSTP2 *promoter shows a pattern of activity similar to that of *NTM19*, but is initiated at tetrad stage [30].

Transcriptomic analyses based on various microarray experiments including those from isolated microspores and developing pollen now provide genome-wide expression profiles throughout plant development [31-33]. Taking advantage of these public databases, we have asked whether microarray data can be directly exploited in order to identify novel and potentially specific promoters that are first active in *Arabidopsis *microspores. In this study, we have selected and characterized the activity of three promoters, *MSP1, MSP2*, and *MSP3 *that were predicted to be specifically active in microspores and developing pollen. We demonstrate that the MSP1 and MSP2 promoters can drive functional protein expression in microspores in complementation experiments and the utility of MSP promoters as novel male-specific microspore expression tools in *Arabidopsis*.

## Results

### Identification of candidate genes

**Figure 1:**
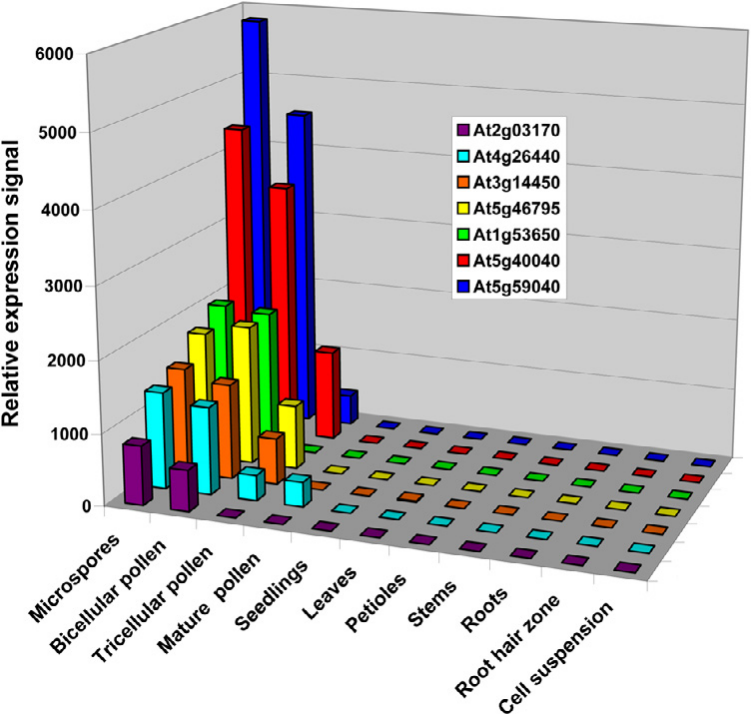
Expression profiles of candidate genes selected for promoter analyses. Expression profiles of seven genes were compared in available male gametophytic and sporophytic transcriptomic datasets. Individual transcriptomic experiments are described in Material and methods.

To identify regulatory sequences that direct preferential or specific expression in *Arabidopsis *microspores we analyzed normalized transcriptomic datasets. We compared the expression profiles of all gametophytically expressed genes with those expressed in the sporophyte. The male gametophyte microarray data set was obtained from our previous experiments involving analysis of four developmental stages; microspore, bicellular, tricellular and mature pollen [33]. Sporophytic datasets were obtained from publicly available resources (NASC). We selected for genes exhibiting strict expression patterns at early stages of male gametophyte development with no or low signals in mature pollen. Genes with expression in inflorescences, flower buds [31,34] and developing flowers [32] were retained as candidate genes, but genes with reliable signals in other sporophytic dataset(s) were excluded. Genes were further selected to retain those encoded as single copy genes and that showed a range of expression levels in microspores. This approach led to the identification of seven potential target genes (At5g40040, At5g59040, At5g46795, At4g26440, At2g03170, At3g14450 and At1g53650; Fig. 1).

**Figure 2:**
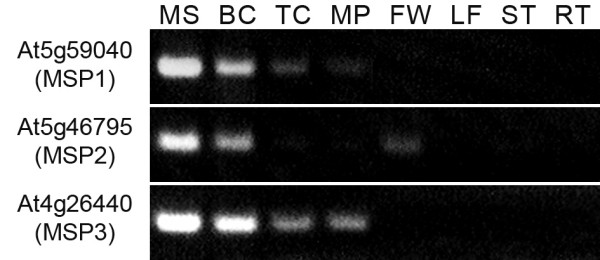
Verification of microarray gene expression data by RT-PCR. The expression of three genes selected for further GUS expression assays was examined in microspores (MS); bicellular (BC), tricellular (TC) and mature pollen (MP); whole flowers (FW); leaves (LF); stems (ST) and roots (RT).

The direct comparison of gene expression levels obtained from independent microarray datasets for male gametophytic cells and for sporophytic tissues is difficult [1,35]. Therefore, the putative specificity of gene expression from microarray analyses was verified by RT-PCR analysis using RNA samples isolated from four stages of male gametophyte development, unicellular, bicellular, tricellular and mature pollen, and four sporophytic tissues and organs (flowers, leaves, stems and roots). Four genes showed expression in one or more sporophytic tissues and were eliminated from further characterization. The remaining three genes showed microspore-specific expression by RT-PCR analysis (Fig. 2). During selection we did not exclude genes from further analysis that also showed weak expression in open flowers since these contain mature pollen grains.

The putative microspore-specific promoter sequences of these genes, At5g59040, At5g46795, At4g26440, were termed MSP1, MSP2 and MSP3 respectively. Selected genes encoded proteins with quite distinct cellular functions. At5g59040 encodes COPT3, a putative pollen-specific member of a copper transporter family [36]. At4g26440 encodes AtWRKY34, a group I WRKY family transcription factor [37]. On the contrary, At5g46795 encodes an expressed protein of unknown function.

### Histochemical analysis of promoter specificity

To test the activity of the selected MSP regulatory sequences *in vivo*, approximately 1 kb upstream genomic fragments were amplified for each of the genes and cloned into the GATEWAY-compatible destination vector, pKGWFS7 [38]. Three MSP-GFP::GUS constructs, MSP1 (At5g59040), MSP2 (At5g46795) and MSP3 (At4g26440), were introduced into wild type *Arabidopsis *plants and ~40 T1 transformants were analyzed histochemically for GUS activity in inflorescences, bud clusters and flowers. We found that 38/40 MSP1, 36/38 MSP2 and 18/34 MSP3 plants showed GUS expression in flower buds and/or in open flowers. GUS staining in MSP1 plants was clearly detectable in anthers of younger buds than those from MSP2 and MSP3, but gradually became weaker towards anthesis, while GUS expression from MSP2 and MSP3 remained high at later stages including in mature pollen grains.

**Figure 3:**
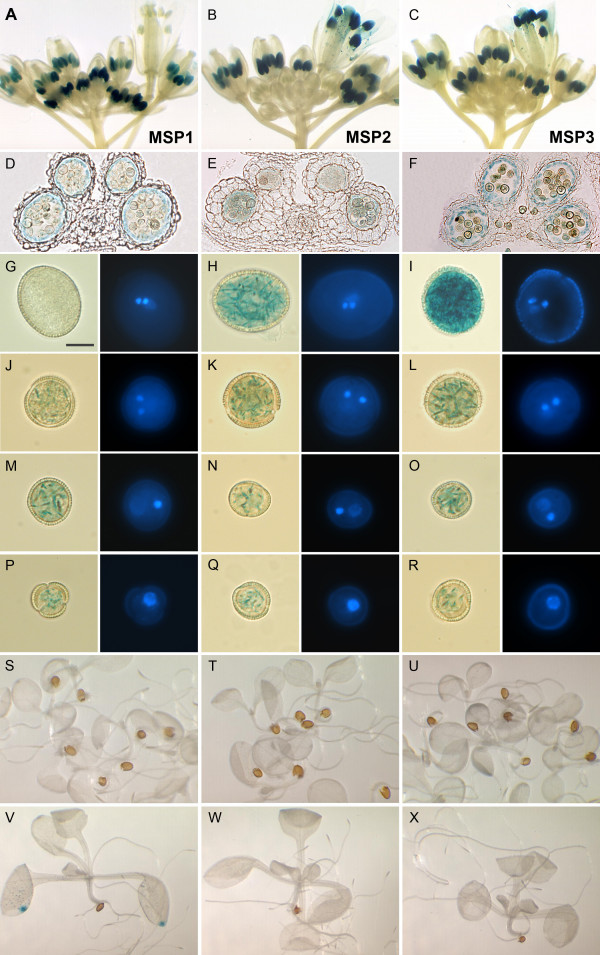
In situ GUS expression driven by three promoters, MSP1 (A, D, G, J, M, P, S and V), MSP2 (B, E, H, K, N, Q, T and W) and MSP3 (C, F, I, L, O, R, U and X). GUS staining in whole inflorescences (A, B, C); transverse sections of whole anthers (D, E, F); five-day (S, T, U) and ten-day old (V, W, X) seedlings. Light and DAPI-stained fluorescence images of mature pollen (G, H, I), immature tricellular pollen (J, K, L) bicellular pollen (M, N, O) and uninucleate microspores (P, Q, R) are shown.

Segregation analysis revealed that the majority of lines segregated 3:1 for kanamycin-resistant and kanamycin-sensitive plants. Plants that were hemizygous for MSP constructs showed 1:1 segregation of GUS staining and non-staining spores consistent with gametophytic expression (data not shown). We examined gametophytic expression in detail by staining isolated spores at different developmental stages and by sectioning stained flower buds. For all three promoters, GUS expression was first detectable in uninucleate microspores (Fig. 3P–R). Another common feature was that anther transverse sections clearly showed GUS staining in the tapetum (Fig 3D–F). Differences in expression profiles were noted between MSP1 and the other two promoters. While MSP1 showed earlier staining in microspores then a strong decline in mature flowers, MSP2 and MSP3 initiate expression in microspores, but GUS expression peaks later and accumulates in mature pollen.

We also examined GUS expression in seedlings using ~20 lines of T2 generation plants for each construct and found no expression from all three MSP promoters, apart from 5–10% of anomalous lines that showed patchy expression in leaves and roots, or weak expression in stamen vascular tissues. None of the lines examined showed GUS activity in 5 day old seedlings. However interestingly, we consistently observed GUS activity at the distal tip of cotyledons and leaves in 10 day old MSP1-GUS seedlings (Fig. 3V). In summary all 3 MSP promoters were found to be specifically or highly preferentially expressed in microspores, developing pollen and the tapetum (Fig. 3)

### Complementation analysis

**Figure 4:**
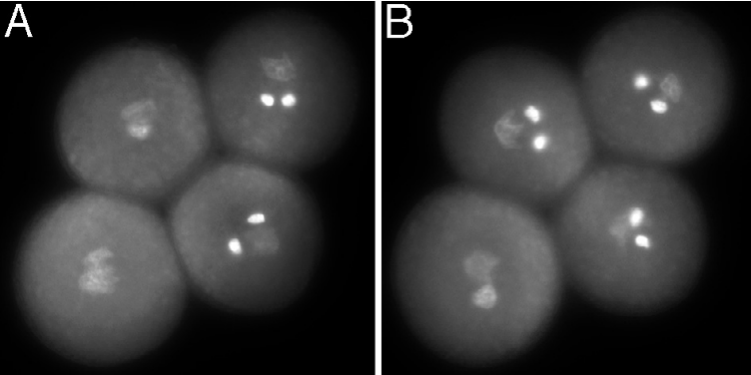
Mature pollen tetrad phenotype after DAPI staining. (A) Tetrad from *+/tio-3;qrt1/qrt1 *plant showing 2:2 segregation of wild type and *tio *mutant pollen. (B) Tetrad from a transformant containing *MSP1-TIO *in the *+/tio-3;qrt1/qrt1 *background, showing three pollen grains with a wild type phenotype and a single mutant *tio *pollen grain.

To evaluate *MSP *promoters as tools for the manipulation of microspore gene expression *in planta*, we examined whether gene expression driven by *MSP1 *and *MSP2 *could complement a gametophytic mutant phenotype caused by defects that are known to depend upon gametophytic expression in developing microspores. Mutations in the *Arabidopsis TWO-IN-ONE *(*TIO*) protein kinase result in binucleate pollen grains due to the failure of cytokinesis in microspores at pollen mitosis I. Plants that are heterozygous for the T-DNA insertion allele, *tio-3*, show 50 % mutant pollen that results in a 2:2 segregation of wild type and mutant pollen in tetrads (Fig. 4A). Moreover cytokinesis defects in mutant *tio-3 *pollen completely block genetic transmission of *tio-3 *through pollen [3].

Test vectors were built in which *MSP1 *or *MSP2 *promoters drive the expression of full length TIO cDNA. pMSP1-TIO and pMSP2-TIO. Both vectors were transformed into *tio-3 *plants by floral dipping. Double selection for *tio-3 *(ppt) and pMSP-TIO (kanamycin) constructs led to the isolation of 19 nineteen transformants containing pMSP1-TIO and 10 containing pMSP2-TIO. Plants were screened for the frequency of wild type and mutant spores in mature pollen tetrads after DAPI staining. 18/19 lines from pMSP1-TIO and 4/10 from pMSP2-TIO showed an increase in the frequency of wild type pollen compared to heterozygous *tio-3 *plants (data not shown). This resulted in the frequent appearance of mature tetrads with three wild type spores and one mutant member, compared with heterozygous *tio-3 *plants that always showed a 2:2 segregation (Fig. 4).

We further tested genetic transmission of the *tio-3 *mutant allele in 15 complementing pMSP1-TIO lines and all of four complementing lines from pMSP2-TIO. In the F1 progenies generated from test crosses in which the pollen donor carried *tio-3 *and pMSP-TIO we observed approximately 30 to 50 % pptR progeny for pMSP1-TIO and 8 to 33 % for pMSP2-TIO. Table 1 shows the results for four representative lines for each construct. These results clearly demonstrate that gametophytic expression of the full-length *TIO *cDNA under the control of *MSP1 *and *MSP2 *promoters is sufficient to complement the *tio *pollen phenotype. Moreover, our results indicate that *MSP1 *is more effective than *MSP2 *in this complementation assay.

## Discussion

We developed a strategy to identify promoters expressed specifically in *Arabidopsis *microspores by exploiting *in silico *analyses and *in vivo *functional analysis. We tested the specificity and timing of three candidate promoters by promoter-GUS fusion analysis. All three promoters were specifically expressed in anthers with the exception of MSP1 that also showed limited expression in the distal tips of cotyledons and true leaves. Within developing flowers their expression was restricted to microspores, developing pollen and tapetal cells. Furthermore, successful use of the MSP1 and MSP2 promoters for complementation of the *tio-3 *mutation demonstrated that both promoters directed functional expression in uninucleate microspores before pollen mitosis I. These promoters therefore provide new tools for the functional analysis of genes and proteins expressed during microspore development. Differences in expression profiles were observed between MSP1, that showed earlier expression and a decline in mature pollen, and MSP2 and MSP3, in which expression increased during pollen maturation. These differences in GUS expression profiles were not predicted by the MSP microarray expression profiles that were very similar. This result and the minor expression patterns observed in MSP1 seedlings highlights the need for experimental verification of specificity prior to further practical analyses.

In developing stamens, cell lineages that lead to male gametophytes and tapetal cells can both be traced to archesporial cells derived from the L2 layer of anther primordial [39]. Moreover, there is strong dependence of microspore development on tapetal cell function. In this regard the co-regulation of gene expression in both microspores and tapetum that occurs at early stages of anther development is not surprising. In tobacco, a chalcone-synthase-like gene ([40] and a chimeric Ca^2+^calmodulin-dependent protein kinase [41]) follow this expression pattern. The *Brassica campestris Bcp1 *gene is also co-expressed in tapetum and microspores [29]. However the corresponding Bgp1 upstream sequences that were active in both tapetum and microspores in *B. campestris *and *Arabidopsis *exhibited pollen-specific expression in tobacco. Therefore different *cis*-acting sequence elements appear to be responsible for coordinated gene expression in tapetum and microspores in *Brassicaceae *and *Solanaceae *families [29]. In *Arabidopsis*, the *ABORTED MICROSPOROGENESIS *(*AMS*) gene encodes a MYC-class transcription factor from the basic helix-loop-helix gene family. AMS is coordinately expressed in tapetum and microspores [42]. Interestingly, the MSP3 gene encodes a member of the WRKY transcription factor family that could also have a role in coordinated gene expression in tapetum and microspores.

## Conclusion

Taken together, we have characterized three *Arabidopsis *microspore-expressed promoters MSP1, MSP2 and MSP3 with early expression profiles specific to the male gametophyte and tapetum. The MSP1 and MSP2 promoters were used successfully to complement cytokinesis functions required to complete microspore development. These tools can be applied to manipulate gene expression in microspores and tapetum without detrimental effects that may arise from undesirable gene expression in other sporophytic tissues.

## Methods

### Plant material and spore isolation

*Arabidopsis *plants were grown in controlled-environment cabinets at 21°C under illumination of 150 mmol m^-2 ^s^-1 ^with a 16-h photoperiod. For spore isolation, *Arabidopsis *ecotype Landsberg erecta (Ler) plants were used. Isolation of spores and pollen was described [33]. Information on the purity of isolated fractions determined by light microscopy and 4,6-diamino-phenylindole staining and vital staining of isolated spore populations assessed by fluorescein-3,6-diacetate treatment were also described in [33]. Roots were grown from plants in liquid cultures as previously described [33]. Wild-type and transgenic seeds were sterilized according to published procedures [43,44]. Plants were transformed by floral dipping [45].

### DNA Chip Hybridization and data normalisation and selection of potential target genes

RNA isolation and hybridization of Affymetrix ATH1 genome arrays was described in [33]. Twelve mixed and seventy-five sporophytic datasets used for comparison with the pollen transcriptome were obtained from the NASCArray database [46] through AffyWatch service [31]. Sporophytic datasets represented seven vegetative tissues (seedlings, leaves, petioles, stems, roots, root hair zone and suspension cell cultures; [33]. Mixed datasets originated from three experiments covering whole inflorescences and flower buds [31,34] and whole flower development [32].

All gametophytic and sporophytic datasets were normalized using DNA-Chip Analyzer 1.3 (dChip) [47] as described previously [33]. All raw and dChip-normalized transcriptomic datasets can be accessed and downloaded through the Arabidopsis Gene Family Profiler (aGFP) database [48].

### RT-PCR analysis

Total RNA from 50 mg of leaves, stems, roots, inflorescences and isolated spores at each developmental stage was extracted using the RNeasy plant kit (Qiagen, Valencia, CA) according to the manufacturer's instructions. The yield and RNA purity were determined spectrophotometrically. Pollen, stem, leaf, and inflorescence samples were isolated from plants grown as described. Pollen RNA used for RT-PCR analyses was obtained from plants that were grown independently from those used to isolate RNA for microarray analysis. Samples of 1 μg total RNA were reverse transcribed in a 20-μL reaction using the ImProm-II Reverse Transcription System (Promega, Madison, WI) following the manufacturer's instructions with the exception that the oligo(dT)_15 _primer was replaced with a custom-synthesized 3'-RACE primer (Tab. 2). The use of intron-spanning primer sets or nested reverse primers in cases where primers spanned no intron ensured that only cDNA was amplified. For PCR amplification, 1 μL of 50× diluted RT mix was used. The PCR reaction was carried out in 25 μL with 0.5 unit of Taq DNA polymerase (MBI Fermentas, Vilnius, Latvia), 1.2 mM MgCl_2_, and 20 pmol of each primer. The PCR program was as follows: 2 min at 95°C, 33 cycles of 15 s at 94°C, 15 s at the optimal annealing temperature (63°C to 67°C), and 30 s at 72°C, followed by 10 min at 72°C. As a reverse primer, NESTED primer (Tab. 2) overlapping the 3'-RACE primer was used to eliminate genomic DNA amplification. The gene-specific forward primers were designed using Primer3 software [49] (Tab. 2).:

### Construction of promoter::GUS reporters

To examine the precise gene expression, each tested gene promoter region was fused with GUS to generate the MSP::GUS reporter using GATEWAY cloning system according to manufacturer's instructions (Invitrogen, Carlsbad, CA). Promoter fragments were amplified by two-step PCR from Col-0 genomic DNA isolated from 3-week-old seedlings using the KOD HiFi DNA Polymerase (Novagen, Darmstadt, Germany). For the first step, specific primers with appended adapters complementary to AttB1 and AttB2 sequences were used to generate 1000-bp promoter regions of tested genes (Tab. 2). Purified PCR products were cloned via pDONR201 donor vector (Invitrogen, Carlsbad, CA) into pKGWFS7 destination vector (VIB, Gent, Belgium; [38] to generate recombinant destination clones pMSP1, pMSP2 and pMSP3. All the recombinant plasmids were transformed into *Agrobacterium tumefaciens *GV3101 [50]. These strains were used to transform *Arabidopsis thaliana *ecotype Columbia using the floral dip method [45]. Transgenic progenies were selected either on one-half strength Murashige and Skoog standard medium, supplemented with 50 mg/l kanamycin on 1-week-old seedlings.

### Histochemical staining of GUS activity

Histochemical assays for GUS activity in T2 generation of Arabidopsis transgenic plants were performed according to the protocol described previously [44,51]. Seedlings and inflorescences incubated for 48 h at 37°C in GUS buffer (100 mM Na-phosphate, pH 7.2; 10 mM EDTA, pH 8.0; 0.1% Triton X-100; 2 mM K_3_Fe [CN]_6_) supplemented with 1 mM 5-bromo-4-chloro-3-indolyl b-D-glucuronide (X-gluc). GUS stained floral buds were fixed in 70% FAA (Formaldehyde/Acetic acid/Ethanol/water, 5/5/63/27, v/v/v/v) for at least 24 h. After washing with 70% ethanol, samples were dehydrated gradually in the ethanol-butanol series and infiltrated with paraffin. 10 μm thin cross sections were prepared with a microtome (Finesse ME, Shandon). GUS staining patterns were recorded using a Nikon Eclipse E600 microscope (Nikon Instruments, Melville, NY). Images were processed using Adobe Photoshop software (version CS2; Adobe Systems, San Jose, CA).

### Complementation analysis

The full-length TIO cDNA clone, pKS-TIONC19, was modified to insert the ~1 kb *MSP1 *or *MSP2 *promoter fragments upstream of the TIO coding sequence using *AscI *and *NotI*. MSP promoter-TIO cDNA fusion fragments were subcloned into the binary vector pER10 [52] using *AscI *and *PacI *to produce pMSP1-TIO and pMSP2-TIO. Before transformation into *Agrobacterium tumefaciens *strain GV3101, constructs were verified by restriction enzyme digestion and sequencing. Individual phosphinothricin (ppt) resistant heterozygous *tio-3 *mutants were transformed by floral dipping [45]. Transformants containing both *tio-3 *and MSP promoter-TIO T-DNA constructs were selected on 15 cm MS agar plates supplemented with 10 mg/l ppt, 50 mg/l kanamycin, and 200 mg/l cefotaxime. Complementation was analyzed by scoring tio-3 pollen phenotype after 4'-6-Diamidino-2-phenylindole (DAPI) staining and also by scoring the genetic transmission of *tio-3 *allele through male after test crosses to Col-0 or *ms1-1 *as described by [2,53].

## Authors' contributions

DH and DT analysed microarray data and selected candidate genes. SAO constructed reporters and carried out the molecular genetic studies. DH and RB verified the gene expression profiles. SAO and JAJ performed complementation analyses. DH, SAO and DT drafted the manuscript. DR, MD, BS and DT carried out the histochemical analysis and imaging. All authors read and approved the final manuscript.

## Figures and Tables

**Table 1:** Complementation analysis

	% mutant pollen	KmR:KmS T2	pptR:pptS F1	% pptR F1
pMSP1-TIO-3	28.7	48:12	30:61	32.96
pMSP1-TIO-9	14.5	103:5	284:294	49.13
pMSP1-TIO-10	11.9	91:10	193:237	44.88
pMSP1-TIO-18	27.9	56:18	43:90	32.33

pMSP2-TIO-1	40.3	323:67	41:145	22.04
pMSP2-TIO-2	43.3	91:15	29:156	15.68
pMSP2-TIO-5	29.2	88:3	29:60	32.58
pMSP2-TIO-8	46.6	85:31	11:131	7.75

*+/tio-3*	50.6	-	0:215	0

Four lines harbouring each construct were compared to the heterozygous parent plant, *tio-3*. The % of mutant pollen was scored after DAPI staining and the T2 segregation of the complementing T-DNA (MSP-TIO) was analysed on kanamycin media. The extent of male transmission (% pptR) of *tio-3 *was determined on ppt media in F1 progenies from test-crosses.

**Table 2:** PCR primers used

Primer	Primer sequence
*cDNA construction*	
3'-RACE	5'-AAGCAGTGGTAACAACGCAGAGTAC(T)_30_VN-3'
*RT-PCR reverse primer*	
NESTED	5'-AAGCAGTGGTAACAACGCAGAGT-3'
*RT-PCR forward primers*	
At5g40040	5'-CTTATCGCTGTTGGACGAGAGAAGA-3'
At5g59040	5'-TCTGTCTCGCCGTCATTTTTGTTAT-3'
At5g46795	5'-GGCTTTGGAGACCAGACTTTTTCAG-3'
At4g26440	5'-TGGAGAGGTAGAAGAGTCCGAATCA-3'
At2g03170	5'-AGAAACACGTCGTTGACGAAGAAAG-3'
At3g14450	5'-GGCCAAGGAGTTTTTCCCTTCTTA-3'
At1g53650	5'-CTCGATCATTGAGCTCAGAAGCTGT-3'.
*Construction of promoter::GUS reporters*	
MSP1-F	5'-AAAAAGCAGGCTTGTCAGTTAGCATGAAAAATTGTATGTTAG-3'
MSP1-R	5'-AGAAAGCTGGGTTTGTTGTGTATACTTGTGTGTGTGTATTTA-3'
MSP2-F	5'-AAAAAGCAGGCTATGTCCTACGATCAGAAGGAGGAG-3'
MSP2-R	5'-AGAAAGCTGGGTAACATGTGATATTATTTTTTTGGTTTATATAGTGG-3'
MSP3-F	5'-AAAAAGCAGGCTTTGTGATATAATAGGTATATATGGTAGAAC-3'
MSP3-R	5'-AGAAAGCTGGGTTGCAAACCCAAGTTTCAGCTTTAAC-3'

Primers used for cDNA synthesis, RT-PCR analyses and construction of promoter::GUS reporters. Where appropriate, appended adapters complementary to AttB1 and AttB2 primers are underlined.
